# Sulfur dioxide in the mid-infrared transmission spectrum of WASP-39b

**DOI:** 10.1038/s41586-024-07040-9

**Published:** 2024-01-17

**Authors:** Diana Powell, Adina D. Feinstein, Elspeth K. H. Lee, Michael Zhang, Shang-Min Tsai, Jake Taylor, James Kirk, Taylor Bell, Joanna K. Barstow, Peter Gao, Jacob L. Bean, Jasmina Blecic, Katy L. Chubb, Ian J. M. Crossfield, Sean Jordan, Daniel Kitzmann, Sarah E. Moran, Giuseppe Morello, Julianne I. Moses, Luis Welbanks, Jeehyun Yang, Xi Zhang, Eva-Maria Ahrer, Aaron Bello-Arufe, Jonathan Brande, S. L. Casewell, Nicolas Crouzet, Patricio E. Cubillos, Brice-Olivier Demory, Achrène Dyrek, Laura Flagg, Renyu Hu, Julie Inglis, Kathryn D. Jones, Laura Kreidberg, Mercedes López-Morales, Pierre-Olivier Lagage, Erik A. Meier Valdés, Yamila Miguel, Vivien Parmentier, Anjali A. A. Piette, Benjamin V. Rackham, Michael Radica, Seth Redfield, Kevin B. Stevenson, Hannah R. Wakeford, Keshav Aggarwal, Munazza K. Alam, Natalie M. Batalha, Natasha E. Batalha, Björn Benneke, Zach K. Berta-Thompson, Ryan P. Brady, Claudio Caceres, Aarynn L. Carter, Jean-Michel Désert, Joseph Harrington, Nicolas Iro, Michael R. Line, Joshua D. Lothringer, Ryan J. MacDonald, Luigi Mancini, Karan Molaverdikhani, Sagnick Mukherjee, Matthew C. Nixon, Apurva V. Oza, Enric Palle, Zafar Rustamkulov, David K. Sing, Maria E. Steinrueck, Olivia Venot, Peter J. Wheatley, Sergei N. Yurchenko

**Affiliations:** 1 Center for Astrophysics | Harvard & Smithsonian, Cambridge, MA USA; 2 Department of Astronomy and Astrophysics, University of Chicago, Chicago, IL USA; 3 Laboratory for Atmospheric and Space Physics, University of Colorado Boulder, Boulder, CO USA; 4 Center for Space and Habitability, University of Bern, Bern, Switzerland; 5 Department of Earth Sciences, University of California, Riverside, Riverside, CA USA; 6 Department of Physics, University of Oxford, Oxford, UK; 7 Institut Trottier de Recherche sur les Exoplanètes, Université de Montréal, Montréal, Quebec Canada; 8 Département de Physique, Université de Montréal, Montréal, Quebec Canada; 9 Department of Physics, Imperial College London, London, UK; 10 Bay Area Environmental Research Institute, NASA Ames Research Center, Moffett Field, CA USA; 11 Space Science and Astrobiology Division, NASA Ames Research Center, Moffett Field, CA USA; 12 School of Physical Sciences, The Open University, Milton Keynes, UK; 13 Earth and Planets Laboratory, Carnegie Institution for Science, Washington, DC USA; 14 Department of Physics, New York University Abu Dhabi, Abu Dhabi, United Arab Emirates; 15 Center for Astro, Particle, and Planetary Physics (CAP3), New York University Abu Dhabi, Abu Dhabi, United Arab Emirates; 16 Centre for Exoplanet Science, University of St Andrews, St Andrews, UK; 17 Department of Physics & Astronomy, University of Kansas, Lawrence, KS USA; 18 Institute of Astronomy, University of Cambridge, Cambridge, UK; 19 Lunar and Planetary Laboratory, University of Arizona, Tucson, AZ USA; 20 Department of Space, Earth and Environment, Chalmers University of Technology, Gothenburg, Sweden; 21 Instituto de Astrofísica de Canarias (IAC), Tenerife, Spain; 22 INAF – Palermo Astronomical Observatory, Palermo, Italy; 23 Space Science Institute, Boulder, CO USA; 24 School of Earth and Space Exploration, Arizona State University, Tempe, AZ USA; 25 Planetary Sciences Section, Jet Propulsion Laboratory, California Institute of Technology, Pasadena, CA USA; 26 Department of Earth and Planetary Sciences, University of California, Santa Cruz, Santa Cruz, CA USA; 27 Centre for Exoplanets and Habitability, University of Warwick, Coventry, UK; 28 Department of Physics, University of Warwick, Coventry, UK; 29 Astrophysics Section, Jet Propulsion Laboratory, California Institute of Technology, Pasadena, CA USA; 30 School of Physics and Astronomy, University of Leicester, Leicester, UK; 31 Leiden Observatory, University of Leiden, Leiden, The Netherlands; 32 INAF – Turin Astrophysical Observatory, Pino Torinese, Italy; 33 Space Research Institute, Austrian Academy of Sciences, Graz, Austria; 34 Space and Planetary Sciences, Institute of Physics, University of Bern, Bern, Switzerland; 35 Université Paris-Saclay, CEA, CNRS, AIM, Gif-sur-Yvette, France; 36 Department of Astronomy, Cornell University, Ithaca, NY USA; 37 Carl Sagan Institute, Cornell University, Ithaca, NY USA; 38 Division of Geological and Planetary Sciences, California Institute of Technology, Pasadena, CA USA; 39 Max Planck Institute for Astronomy, Heidelberg, Germany; 40 SRON Netherlands Institute for Space Research, Leiden, The Netherlands; 41 Université Côte d’Azur, Observatoire de la Côte d’Azur, CNRS, Laboratoire Lagrange, French Riviera, France; 42 Department of Earth, Atmospheric and Planetary Sciences, Massachusetts Institute of Technology, Cambridge, MA USA; 43 Kavli Institute for Astrophysics and Space Research, Massachusetts Institute of Technology, Cambridge, MA USA; 44 Astronomy Department, Wesleyan University, Middletown, CT USA; 45 Van Vleck Observatory, Wesleyan University, Middletown, CT USA; 46 Johns Hopkins University Applied Physics Laboratory, Laurel, MD USA; 47 School of Physics, University of Bristol, Bristol, UK; 48 Indian Institute of Technology Indore, Indore, India; 49 Department of Astronomy and Astrophysics, University of California, Santa Cruz, Santa Cruz, CA USA; 50 NASA Ames Research Center, Moffett Field, CA USA; 51 Department of Astrophysical and Planetary Sciences, University of Colorado Boulder, Boulder, CO USA; 52 Department of Physics and Astronomy, University College London, London, UK; 53 Instituto de Astrofisica, Facultad Ciencias Exactas, Universidad Andres Bello, Santiago, Chile; 54 Centro de Astrofisica y Tecnologias Afines (CATA), Santiago, Chile; 55 Núcleo Milenio de Formación Planetaria (NPF), Valparaíso, Chile; 56 Anton Pannekoek Institute for Astronomy, University of Amsterdam, Amsterdam, The Netherlands; 57 Planetary Sciences Group, Department of Physics and Florida Space Institute, University of Central Florida, Orlando, FL USA; 58 Institute of Planetary Research, German Aerospace Center (DLR), Berlin, Germany; 59 Department of Physics, Utah Valley University, Orem, UT USA; 60 Department of Astronomy, University of Michigan, Ann Arbor, MI USA; 61 Department of Physics, University of Rome “Tor Vergata”, Rome, Italy; 62 Universitäts-Sternwarte, Ludwig-Maximilians-Universität München, München, Germany; 63 Exzellenzcluster Origins, Garching, Germany; 64 Department of Astronomy, University of Maryland, College Park, MD USA; 65 Department of Earth and Planetary Sciences, Johns Hopkins University, Baltimore, MD USA; 66 Department of Physics and Astronomy, Johns Hopkins University, Baltimore, MD USA; 67 Université de Paris Cité and Université Paris-Est Creteil, CNRS, LISA, Paris, France

**Keywords:** Exoplanets, Atmospheric chemistry

## Abstract

The recent inference of sulfur dioxide (SO_2_) in the atmosphere of the hot (approximately 1,100 K), Saturn-mass exoplanet WASP-39b from near-infrared JWST observations^1–3^ suggests that photochemistry is a key process in high-temperature exoplanet atmospheres^4^. This is because of the low (<1 ppb) abundance of SO_2_ under thermochemical equilibrium compared with that produced from the photochemistry of H_2_O and H_2_S (1–10 ppm)^4–9^. However, the SO_2_ inference was made from a single, small molecular feature in the transmission spectrum of WASP-39b at 4.05 μm and, therefore, the detection of other SO_2_ absorption bands at different wavelengths is needed to better constrain the SO_2_ abundance. Here we report the detection of SO_2_ spectral features at 7.7 and 8.5 μm in the 5–12-μm transmission spectrum of WASP-39b measured by the JWST Mid-Infrared Instrument (MIRI) Low Resolution Spectrometer (LRS)^10^. Our observations suggest an abundance of SO_2_ of 0.5–25 ppm (1*σ* range), consistent with previous findings^4^. As well as SO_2_, we find broad water-vapour absorption features, as well as an unexplained decrease in the transit depth at wavelengths longer than 10 μm. Fitting the spectrum with a grid of atmospheric forward models, we derive an atmospheric heavy-element content (metallicity) for WASP-39b of approximately 7.1–8.0 times solar and demonstrate that photochemistry shapes the spectra of WASP-39b across a broad wavelength range.

## Abstract (web summary)

Observations from the JWST MIRI/LRS show the detection of SO_2_ spectral features in the 5–12-μm transmission spectrum of the hot, Saturn-mass exoplanet WASP-39b, suggesting that photochemistry is a key process in high-temperature exoplanet atmospheres.

## Main

We observed WASP-39b using JWST MIRI/LRS on 14 February 2023 from 15:03:20 UTC to 22:59:36 UTC, spanning a total of 7.94 h (Director’s Discretionary Time PID 2783). The observation included the full 2.8-h transit, as well as 3 h before and 1.87 h after the transit to measure the stellar baseline. We used the slitless prism mode with no dithering. In this mode, MIRI/LRS yields a spectral range from 5 to 12 μm, at an average resolving power of *R* ≡ *λ*/Δ*λ* ≈ 100, in which *λ* is the wavelength. The time-series observations included 1,779 integrations of 16 s (100 groups per integration). No region of the detector was saturated.

**Fig. 1:**
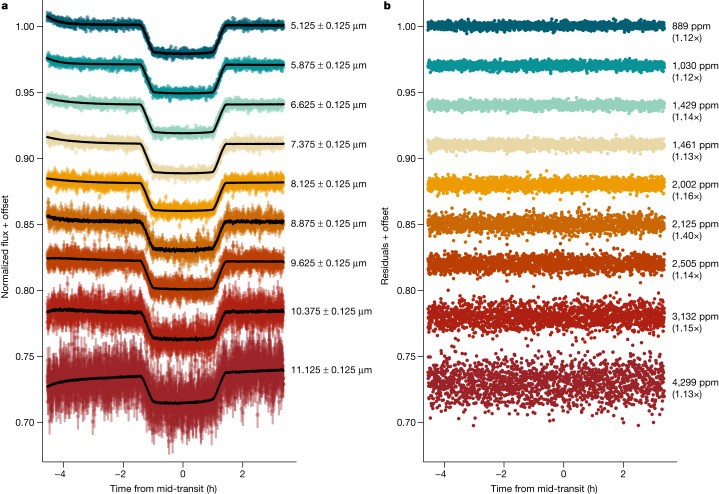
A sample of spectrophotometric light curves and residuals for the transit of WASP-39b observed with MIRI/LRS. **a**, An exoplanet transit model multiplied by a systematics model (solid black line) was fitted to each light curve. **b**, The residuals to the best-fit models are shown for each light curve. We report the 1*σ* scatter in each light curve as the standard deviation of the out-of-transit residuals, with the ratio to the predicted photon noise in parentheses. The reduction is from Eureka!.

**Extended Data Fig. 1:**
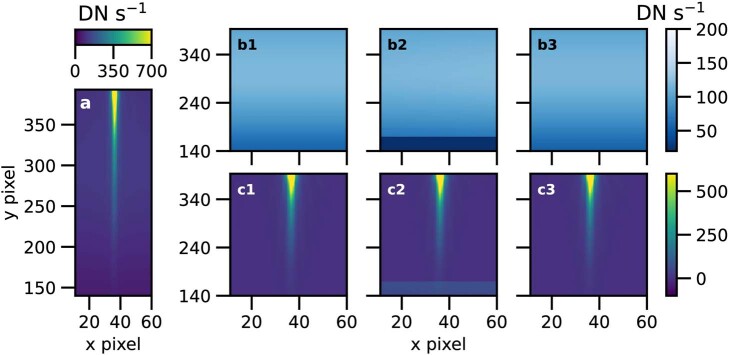
Comparison of the different background modelling and subtraction per each pipeline. **a**, A median out-of-transit image of the MIRI/LRS detector from the jwst pipeline’s Stage 2 processing. **b**, Background models from Eureka! (1), Tiberius (2) and SPARTA (3). **c**, Background-subtracted Stage 2 outputs from each pipeline. The smoothly varying background is expected for MIRI/LRS. There are no discrete features or sharp changes in the background at *y* pixels < 244, corresponding to *λ* = 10 μm, which has been seen in other observations^13^. All images are given in Data Numbers per second (DN s^−1^). The Tiberius reduction did not extract spectra as far red as Eureka! and SPARTA, which is the cause of the horizontal bar in panels **b2** and **c2**.

**Extended Data Fig. 2:**
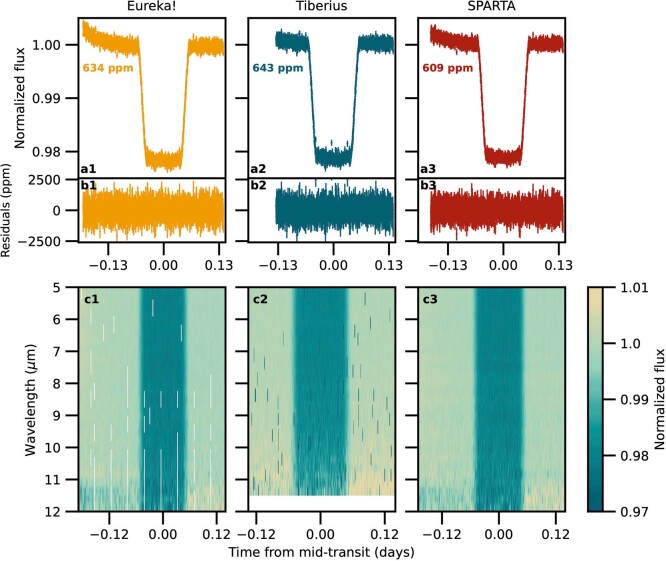
MIRI/LRS white and spectrophotometric light curves from the three independent reduction pipelines used in this work. **a**, We quote the out-of-transit ppm scatter in each light curve in the figure. We define the out-of-transit time as −0.135 < *t* (days) < −0.07 and 0.07 < *t* (days) < 0.14; these times were selected as they ignore the exponential ramp at the beginning of the observations and do not include any data in transit ingress/egress. **b**, The residuals and errors of the data compared with the best-fit transit model. Errors quoted are 1*σ*. **c**, The spectrophotometric light curves are normalized by the out-of-transit flux during the observations. All reductions show consistent out-of-transit scatter in all wavelength bins (Δ*λ* = 0.25 μm). The white spaces in **c1** are where values in the light curve are NaN.

We extracted the time-series stellar spectra using three independently developed reduction pipelines to test the impact of background modelling, spectral extraction method and aperture width and light-curve-fitting routines on the resulting planetary transmission spectrum (see Methods and Extended Data Figs. 1 and 2). We summed across the extracted stellar spectra to create white-light curves (Extended Data Fig. 2), as well as binned spectrophotometric light curves for each pipeline (Fig. 1). The light curves show clear instrumental systematics at the beginning of the observation that are driven by a decreasing exponential ramp effect^11^. At the detector level, the observations showed correlations with spatial position and an odd–even effect from row to row owing to the readout time^12^. We do not see evidence of a very sharp, strong change in the sign, amplitude or timescale of the initial exponential ramp, known as a ‘shadowed region’, in our observations^13^ (Extended Data Fig. 1). We use wide spectrophotometric-light-curve bins of Δ*λ* = 0.25 μm to average over the odd–even row effect^13^ and note that our conclusions are insensitive to the chosen bin size (smaller bins of 0.15 μm derive the same results) as well as the choice of the origin binning wavelength.

**Fig. 2:**
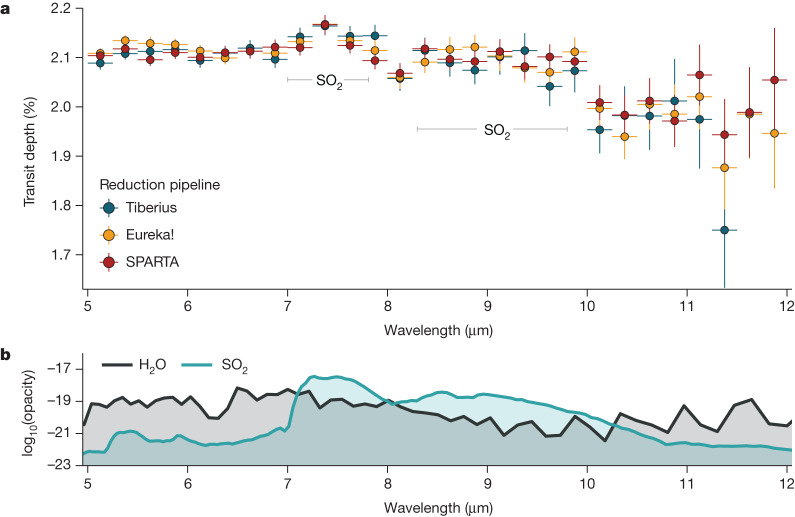
MIRI/LRS transmission spectra of WASP-39b derived using three independent reduction pipelines. **a**, The spectrum is dominated by broad absorption features from SO_2_ at 7.7 and 8.5 μm and H_2_O across the entire wavelength coverage of MIRI/LRS. We define our uncertainties as 1*σ*. **b**, We present the log of opacities of dominant species in the spectrum in units of cm^2^ mol^−1^. The opacities were adopted from PLATON using ExoMol line lists^22,23^ and assume atmospheric properties pressure, *P* = 1 mbar, and temperature, *T* = 1,000 K.

We present the resulting transmission spectrum from each pipeline in Fig. 2. Within the spectra, we are able to identify two broad absorption features belonging to SO_2_ at 7.7 and 8.5 μm, which correspond to the asymmetric *ν*_3_ and symmetric *ν*_1_ fundamental bands, respectively, consistent with predictions from photochemical models^4^. We are also able to discern H_2_O absorption, although it is mostly apparent between 5 and 7 μm owing to the overlapping SO_2_ feature at longer wavelengths. There is an abrupt decrease in the transit depth at *λ* = 10 μm. The shadowed region systematic occurs from *λ* ≥ 10.6–11.8 μm (ref. ^13^), at longer wavelengths compared with the abrupt decrease in the transmission spectrum. Therefore, if this abrupt change arose from the instrument and is not of astrophysical origin, then it is most likely driven by a different source of detector noise or an artefact that is not well understood at present.

**Fig. 3:**
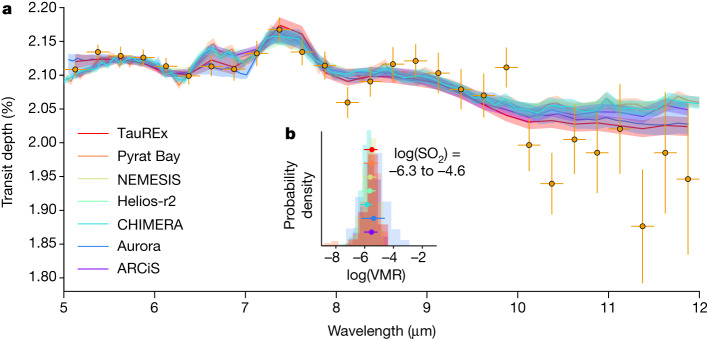
Free retrievals of the MIRI/LRS transmission spectrum of WASP-39b. **a**, The spectrum from the Eureka! reduction (with 1*σ* uncertainties) is compared with the best-fit retrieved spectra and associated 1*σ* shaded regions from six free-retrieval codes. **b**, The corresponding posterior probability distributions of the volume mixing ratio (VMR) and associated 1*σ* uncertainties (points) for the SO_2_ abundance. The quoted log(SO_2_) ranges from the lowest to the highest 1*σ* bounds of all six posteriors. We chose the Eureka! reduction owing to its similar reduction steps to previous WASP-39b observations^2,3,15,16^ and the fact that it provides the full-wavelength coverage of the observations. Results from the other two reductions for SO_2_ give broadly consistent results and are discussed further in Methods.

To determine the detection significance of SO_2_ in our data and constrain its abundance, we conducted seven independent Bayesian retrievals on each of the three data reductions. Each nominal retrieval includes SO_2_ and H_2_O as spectrally active gases, as well as a variety of cloud and haze treatments to account for degeneracies between retrieved cloud/haze properties and molecular abundances (see Methods). Other spectrally active gases were initially tested by the retrievals, including CH_4_, NH_3_, HCN, CO, CO_2_, C_2_H_2_ and H_2_S, but none of them showed significant detections. As shown in Fig. 3 and Extended Data Table 4, the fits of the retrieval models to the data are generally good, with reduced chi-squared values close to 1. SO_2_ is detected to at least approximately 3*σ* significance for all retrieval frameworks and data reductions, except for one single retrieval–data reduction combination with a 2.5*σ* detection, in which other free parameters slightly reduced the SO_2_ detection significance (see Methods). We retrieve a range of log volume mixing ratios from −6.3 to −4.6 (0.5–25 ppm; lowest to highest 1*σ* uncertainty bounds across all six retrieval frameworks) for the Eureka! reduction. Retrievals for the other reductions yielded similar results and are discussed in Methods and shown in Extended Data Fig. 4.

Similar to SO_2_, the retrieved H_2_O abundances are largely consistent across all retrievals and reductions (see Extended Data Table 4 and Extended Data Fig. 4), although the spread of values for the detection significance is greater than for SO_2_, with some reduction–retrieval combinations yielding ≲2*σ*, whereas for others, it is above 5*σ*. This serves to highlight the impact of choices made at both the reduction and retrieval stages on conclusions drawn from a spectrum. We postulate that the variation in detection significance that we see is because of the fact that the H_2_O feature present in this observation is fairly broad, and probably affected by the stronger SO_2_ feature at longer wavelengths and modelled haze properties at shorter wavelengths. For the Aurora/Eureka! combination, the water abundance is relatively poorly constrained, with long tails in the distribution towards lower abundances and haze compensating for the relative lack of H_2_O absorption at short wavelengths. Across the other six retrievals for the Eureka! reduction, the retrieved range of log volume mixing ratios is from −2.4 to −1.2 (0.4–6.3%; lowest to highest 1*σ* uncertainty).

As well as SO_2_ and H_2_O, one retrieval framework found weak to moderate (2.5*σ*) evidence for SO, with a feature between 8 and 10 μm (see Methods), which is predicted to be present by photochemical models^4,5^, but further observations would be needed to confirm or rule out its existence. Furthermore, we can largely rule out a grey cloud extending to low pressures with broad terminator coverage (see Methods), but more detailed cloud and haze properties such as particle sizes and cloud-top pressure cannot be consistently constrained.

**Fig. 4:**
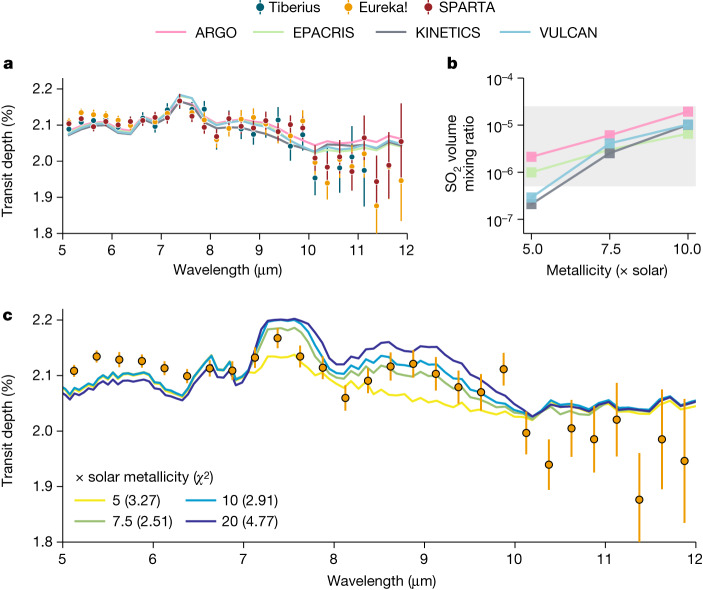
Comparison of four independent photochemical models with the observed MIRI/LRS transmission spectra of WASP-39b. **a**, Comparison of morning and evening limb-averaged theoretical transmission spectra to the observations assuming a best-fit atmospheric metallicity of 7.5 times solar. **b**, Limb-averaged SO_2_ volume mixing ratio between 10 and 0.01 mbar as a function of metallicity for the four photochemical models. The shaded region represents the 1*σ* SO_2_ constraint from the free retrievals on the Eureka! reduction (Fig. 3). **c**, Dependence of VULCAN modelled transmission spectrum on atmospheric metallicity, as compared with the Eureka! reduction. The Tiberius reduction prefers a metallicity of 7.5 times solar, whereas the SPARTA reduction prefers 10 times solar (see Extended Data). The VULCAN models suggest that there is only a minor (<0.05%) difference expected for the SO_2_ feature at 7.7 μm when assuming a higher atmospheric metallicity, whereas the SO_2_ feature at 8.5 μm is more sensitive to subtle changes. The SO_2_ feature at 8.5 μm is fit well by the 7.5–10 times solar metallicity models.

We use a suite of independent forward-model grids that include photochemistry to infer the atmospheric metallicity and elemental ratios of WASP-39b from the observed SO_2_ abundance (see Methods). As SO_2_ is photochemical in origin, a rigorous treatment of photochemistry is vital for connecting SO_2_ to bulk atmospheric properties. Figure 4 shows the comparison between four independent photochemical models, all of which include moderately different chemical networks for H, C, O, N and S molecules and use the same average atmospheric temperature–pressure profiles (morning and evening terminators), eddy-diffusion profile and stellar spectrum of WASP-39 adopted in ref. ^4^ as inputs. The model transmission spectra generated from the four photochemical models are largely consistent with each other and the data, showing that sufficient SO_2_ is generated photochemically to explain the 7.7-μm and 8.5-μm absorption features. In particular, the limb-averaged volume mixing ratio of SO_2_ for the best-fitting 7.5 times solar metallicity models span the range 2.5–6.1 ppm, in line with our free-retrieval results (Extended Data Table 4). The 8.5-μm SO_2_ feature is notably sensitive to metallicity in this range, whereas the strongest 7.7-μm feature starts to saturate with metallicity ≳7.5 times solar.

Using an expanded grid of one of the photochemical models^14^ (see Methods), we find best-fitting atmospheric metallicity values of 7.1–8.0 times solar across the three data reductions, as well as a consistent—although weak—preference for a super-solar O/S ratio, sub-solar C/O and approximately solar C/S. Even though no carbon species is detected in the spectrum, constraints on the carbon abundance are still possible through the high degree of coupling between the CHONS elements in the photochemistry. These results are largely corroborated by comparisons with independent, self-consistent, radiative–convective–thermochemical equilibrium model grids that are post-processed to include SO_2_ (see Methods), which also infer a sub-solar C/O, as well as slightly higher atmospheric metallicity values ranging between 10 and 30 times solar, depending on the specific data reduction. These findings are within the range of C/O (sub-solar) and atmospheric metallicities (super-solar) derived from near-infrared JWST transmission spectra of WASP-39b using self-consistent radiative–convective–thermal equilibrium grid models^1–3,15,16^ and photochemical models that were able to match the near-infrared SO_2_ feature^4^. Our work therefore shows that JWST’s MIRI/LRS is fully capable of producing information-rich exoplanet observations such as those of the near-infrared instruments.

The interpretation of WASP-39b’s transmission spectrum at wavelengths beyond 10 μm is uncertain. If the observed sudden drop in transit depth is astrophysical in origin rather than because of an artefact in the data, then several possibilities exist. For example, the transit radius of a planet can decrease quickly with increasing wavelength when a cloud layer becomes sufficiently optically thin such that we can investigate below the cloud base^17^. Also, spectral features associated with the vibrational modes of bonds of several cloud and haze species are situated in the mid-infrared^18–20^, but none of the known features can explain our data. Meanwhile, the absorption cross-sections of some gaseous species, such as metal hydrides (for example, SiH and BeH), can exhibit downward slopes starting at roughly 10 μm (ref. ^21^). However, the abundances of these species needed to explain the observed feature (about 1,000 ppm) are orders of magnitude greater than what is expected in a near-solar metallicity atmosphere (see Methods). Further observations will be needed to explore the behaviour and provenance of the >10-μm transmission spectrum of WASP-39b.

## Methods

### Data reduction

We applied three independent data-reduction and light-curve-fitting routines to the MIRI/LRS observations. Below, we describe the main reduction steps taken by each pipeline, followed by their light-curve-fitting methodologies. Furthermore, we discuss the differences in the data-reduction pipelines that resulted in differing shapes of the H_2_O absorption feature at <7 μm.

#### Eureka!

Initially, nine independent teams performed a reduction of these data using the open-source Eureka!^24^ pipeline. From those analyses, we ultimately chose one analysis to highlight in this paper based on comparisons of the white and red noise of the residuals after fitting. Our fiducial Eureka! reduction very closely followed the methods developed for the Transiting Exoplanet Early Release Science (ERS) Team’s MIRI/LRS phase-curve observations of WASP-43b and described in refs. ^13,25^. As extensive parameter studies were performed on Eureka!’s Stage 1–3 parameters using the WASP-43b data, the best parameter settings identified from that work are reused here and are briefly summarized below. The other Eureka! analyses had used different reduction parameters and were generally consistent with, but noisier than, our fiducial Eureka! analyses. The full Eureka! Control Files and Eureka! Parameter Files used in these analyses are available as part of the data products associated with this work (10.5281/zenodo.10055845).

We made use of version 0.9 of the Eureka!^24^ pipeline, CRDS version 11.16.16 and context 1045, and jwst package version 1.8.3 (ref. ^26^). As described in refs. ^13,25^, we assume a constant gain of 3.1 electrons per Data Number (DN) (the same as for the SPARTA reduction; see below), which is closer to the true gain than the value of 5.5 assumed in the CRDS reference files at present (private communication, Sarah Kendrew). Eureka!’s Stage 1 jump step’s rejection threshold was increased to 7.0 and Stage 2’s photom step was skipped (to more easily estimate the expected photon noise), but otherwise the Stage 1–2 processing was done following the default settings of the jwst pipeline. We also evaluated the use of an experimental nonlinearity reference file developed to address MIRI’s ‘brighter-fatter effect’^27^, but we ultimately decided to stick with the default nonlinearity reference file, as the final transmission spectra changed by less than 1*σ* at all wavelengths.

We extracted columns 11–61 and rows 140–393, as pixels outside this range are excessively dominated by noise. We masked pixels marked as ‘DO_NOT_USE’ in the DQ array to remove bad pixels identified by the jwst pipeline. To aid in decorrelating systematic noise, we compute a single centroid and point spread function (PSF) width for each integration by summing along the dispersion direction and fitting a 1D Gaussian; only the centroid of the first integration was used to determine aperture locations. We subtracted the background flux by subtracting the mean of pixels separated from the source by 11 or more pixels after first sigma-clipping 5*σ* outliers along the time axis and along the spatial axis. We then performed optimal spectral extraction^28^ using the pixels within 5 pixels of the centroid. Our spatial profile was a cleaned median frame, following the same sigma-clipping methods described in refs. ^13,25^. We then spectrally binned the data into 28 bins, each 0.25 μm wide, spanning 5–12 μm as well as a single white-light curve spanning the full 5–12 μm. To remove any remaining cosmic rays or the effects of any high-gain antenna moves, we then sigma-clipped each light curve, removing any points 4*σ* or more discrepant with a smoothed version of the light curve computed using a boxcar filter with a width of 20 integrations. This removed errant points while ensuring not to clip the transit ingress or egress.

When fitting, our astrophysical model consisted of a starry^29^ transit model with uninformative priors on the planet-to-star radius ratio and unconstrained, reparameterized quadratic limb-darkening parameters^30^. We also used broad priors on the orbital parameters of the planet to verify that these new data are consistent with the orbital solution presented in A.L.C. et al., manuscript in preparation. Specifically, we used Gaussian priors for the transit time, inclination and scaled semimajor axis based on the values in A.L.C. et al., manuscript in preparation, which were derived by fitting all previous WASP-39b observational datasets at once (see values in Extended Data Table 1), but with greatly inflated uncertainties (roughly 10 times or higher than the precision achievable with these MIRI data alone) to allow these data to independently verify the previously published values (A.L.C. et al., manuscript in preparation). We also assumed zero eccentricity and fixed the orbital period to the value of $4.0552842{\pm }_{0.0000035}^{0}\,{\rm{days}}$ from A.L.C. et al., manuscript in preparation. We linearly decorrelated against the changing spatial position and PSF width computed during Stage 3. We also allowed for a linear trend in time as well as a single, weakly constrained exponential ramp to remove the well-known ramp at the beginning of MIRI/LRS observations^11,13,25^. We also trimmed the first ten integrations, as they suffered from a particularly strong exponential ramp. There was no evidence for mirror tilts^31^ in the observations nor any residual impacts from high-gain antenna moves after sigma-clipping the data in Stage 4. Finally, we also used a noise multiplier to capture any excess white noise and ensure a reduced chi-squared of 1. We then used PyMC3’s No-U-Turn Sampler^32^ to sample our posterior. We used two independent chains and used the Gelman–Rubin statistic^33^ to ensure that our chains had converged ($\widehat{R} < 1.01$), and then we combined the samples from the two chains and computed the 16th, 50th and 84th percentiles of the 1D marginal posteriors to estimate the best-fit value and uncertainty for each parameter.

As our determined orbital parameters were consistent with those determined in A.L.C. et al., manuscript in preparation, we then fixed our orbital parameters to those of A.L.C. et al., manuscript in preparation for our spectroscopic fits ensuring consistency with other JWST spectra for this planet. The limb-darkening parameters for our spectroscopic fits were given a Gaussian prior of ±0.1 with respect to model-predicted limb-darkening coefficient spectra^34,35^ based on the Stagger-grid^36^. We also evaluated more conservatively trimming the first 120 integrations (instead of ten) for our spectroscopic fits, but found that the resulting spectra were changed by much less than 1*σ* at all wavelengths.

For our white-light-curve fit, we found a white-noise level 26% larger than the estimated photon limit, whereas the spectroscopic channels were typically 10–20% larger than the estimated photon limit. As our adopted gain of 3.1 is only accurate to within about 10% of the true gain^13,25^ (which varies as a function of wavelength; private communication, Sarah Kendrew), these comparisons with estimated photon limits only give general ideas of MIRI’s performance. An examination of our Allan variance plots^37^ showed minimal red noise in our residuals. Our decorrelation against the spatial position and PSF width showed that the shortest wavelengths were most strongly affected by changes in spatial position and PSF width, with both driving noise at the level of about 100 ppm in the shortest-wavelength bin; meanwhile, the impact at longer wavelengths was weaker and not as well constrained. The orbital parameters determined from the white-light-curve fit are summarized in Extended Data Table 1.

#### Tiberius

Tiberius is a pipeline to perform spectral extraction and light-curve fitting, which is derived from the LRG-BEASTS pipeline^38–40^. It has been used in the analysis of JWST data from the ERS Transiting Exoplanet Community programme and GO programmes^1–3,41^.

In our reduction with Tiberius, we first ran STScI’s jwst pipeline on the uncal.fits files. We performed the following steps in the jwst pipeline: group_scale, dq_init, saturation, reset, linearity, dark_current, refpix, ramp_fit, gain_scale, assign_wcs and extract_2d. Our spectral extraction was run on the gainscalestep.fits files and we used the extract2d.fits files for our wavelength calibration. As explained in the jwst documentation, the gain_scale step is actually benign if the default gain setting is used. For that reason, the Tiberius reduction used units of DN s^−1^. Ultimately, because we normalize our light curves and rescale the photometric uncertainties during light-curve fitting, the units of the extracted stellar flux do not affect the transmission spectrum.

We did not perform the jump or flat_field steps. Instead of the jump step, we performed outlier detection for every pixel in the time series by locating integrations for which a pixel deviated by more than 5*σ* from the median value for that pixel. Any outlying pixels in the time series were replaced by the median value for that pixel. Next we performed spectral extraction. We first interpolated the spatial dimension of the data onto a new grid with ten times the resolution, which improves flux extraction at the sub-pixel level. The spectra were then traced using Gaussians fitted to every pixel row from row 171 to 394. The means of these Gaussians were then fitted with a fourth-order polynomial. We then performed standard aperture photometry at every pixel row after subtracting a linear polynomial fitted across two background regions on either side of the spectral trace. We experimented with the choice of aperture width and background width to minimize the noise in the white-light curve. The result was an 8-pixel-wide aperture and two 10-pixel-wide background regions offset by 8 pixels from the extraction aperture.

Next we cross-correlated the stellar spectrum of each integration with a reference spectrum to measure drifts in the dispersion direction. The reference spectrum was taken to be the 301st integration of the time series, as we clipped the first 300 integrations (80 min) to remove the ramp seen in the transit light curve. The measured shifts had a root mean square of 0.002 pixels in the dispersion direction and 0.036 pixels in the spatial direction (as measured from the tracing step). Next we integrated our spectra in 25 × 0.25-μm-wide bins from 5 to 11.25 μm to make our spectroscopic light curves.

We fitted our light curves with an analytic transit light curve, implemented in batman^42^, multiplied by a time trend. For the white-light curve, this time trend was a quadratic polynomial, as a linear trend was not sufficient. This differed to the other reductions that treated the systematics as exponential ramps with a linear trend. For the spectroscopic light curves, we divided each spectroscopic light curve by the best-fitting transit and systematics model from the white-light-curve fit. A quadratic trend was not necessary for the spectroscopic light curves, which we instead fit with a linear trend to account for residual chromatic trends not accounted for by the common mode correction.

In all light-curve fits, we used Markov chain Monte Carlo implemented using emcee^43^. We set the number of walkers equal to ten times the number of free parameters and ran two sets of chains. The first set of chains was used to rescale the photometric uncertainties to give ${\chi }_{\nu }^{2}=1$ and the second set of chains was run with the rescaled uncertainties. In both cases, the chains were run until they were at least 50 times the autocorrelation length for each parameter. This led to chains between 4,000 and 10,000 steps long.

Given the nonlinear ramp at the beginning of the observations, we clipped the first 300 integrations. We found that this clipping led to a consistent and more precise transmission spectrum. In tests without clipping any integrations, we found that a fifth-order polynomial was needed to fit the ramp. We disfavoured this owing to the extra free parameters. For the white-light curve, our fitted parameters were the time of mid-transit (*T*_0_), orbital inclination of the planet (*i*), semimajor axis scaled by the stellar radius (*a*/R_*_), planet-to-star radius ratio (*R*_P_/R_*_), the three parameters defining the quadratic-in-time polynomial trend and the quadratic limb-darkening coefficients reparameterized following ref. ^30^ (*q*_1_ and *q*_2_). For *q*_1_ and *q*_2_, we used Gaussian priors with means set by calculations from Stagger 3D stellar atmosphere models^34–36^ and standard deviations of 0.1. The period was fixed to 4.0552842518 days, as found from the global fit to the near-infrared JWST datasets (A.L.C. et al., manuscript in preparation). Our best-fitting values for the system parameters are given in Extended Data Table 1.

For our spectroscopic light curves, we fixed the system parameters (*a*/R_*_, *i* and *T*_0_) to the values from the global fit to the near-infrared JWST datasets (A.L.C. et al., manuscript in preparation). The median root mean square of the residuals from the white-light and spectroscopic light-curve fits were 573 and 3,034 ppm, respectively.

#### SPARTA

SPARTA (the Simple Planetary Atmosphere Reduction Tool for Anyone) is an open-source code intended to be simple, fast, bare-bones and utilitarian. SPARTA is fully independent and uses no code from the JWST pipeline or any other pipeline. It was initially written to reduce the MIRI phase curve of GJ 1214b and is described in detail in that paper^44^. SPARTA was also used to reduce the MIRI phase curve of WASP-43b, taken as part of the ERS programme^13,25^. Having learned many best practices from these previous reductions, we performed virtually no parameter optimization for the current WASP-39b reduction. Below, we briefly summarize the reduction steps, but we refer the reader to the previous two papers for more details.

In stage 1, SPARTA starts with the uncalibrated files and performs nonlinearity correction, dark subtraction, up-the-ramp fitting and flat correction, in that order. The up-the-ramp fit discards the first five groups and the last group, which are known to be anomalous, and optimally estimates the slope using the remaining groups by taking the differences between adjacent reads and computing the weighted average of the differences. The weights are calculated with a mathematical formula that gives the optimal estimate of the slope^44^.

After stage 1, SPARTA computes the background by taking the average of columns 10–24 and 47–61 (inclusive, zero-indexed) of each row in each integration. The background is then subtracted from the data. These two windows are equally sized and equidistant from the trace on either side, so any slope in the background is naturally subtracted out.

Next we compute the position of the trace. We compute a template by taking the pixel-wise median of all integrations. For each integration, we shift the template (through bilinear interpolation) and scale the template (through multiplication by a scalar) until it matches the integration. The shifts that result in the lowest *χ*^2^ are recorded.

The aforementioned template, along with the positions we find, are used for optimal extraction. We divide the template by the per-row sum (an estimate of the spectrum) to obtain a profile and shift the profile in the spatial direction by the amount found in the previous step. The shifted profile is then used for optimal extraction, using the algorithm in ref. ^28^. We apply this algorithm only to an 11-pixel-wide (full-width) window centred on the trace and iteratively reject >5*σ* outliers until convergence.

After optimal extraction, we gather all the spectra and the positions into one file. We reject outliers by creating a white-light curve, detrending it with a median filter and rejecting integrations more than 4*σ* away from 0. Sometimes, only certain wavelengths of an integration are bad, not the entire integration. We handle these by detrending the light curve at each wavelength, identifying 4*σ* outliers and replacing them with the average of their neighbours on the time axis.

Finally, we fit the white-light and spectroscopic light curves using emcee. The spectroscopic bins are exactly the same as for the Eureka! and Tiberius reductions: 0.25 μm wide and ranging from 5.00–5.25 μm to 11.75–12.00 μm. We trim the first 112 integrations (30 min) and reject >4*σ* outliers. In the white-light fit, limb-darkening parameters *q*_1_ and *q*_2_ are both free and given broad uniform priors. In the spectroscopic fit, *T*_0_, *P*, *a*/*R*_s_, *b* and the limb-darkening coefficients are fixed to the fiducial values, but the transit depth and the systematics parameters are free. The systematics model is given by

1 $$S={F}_{* }\left(1+A\,\exp (-t/\tau )+{c}_{y}\,y+{c}_{x}\,x+m(t-\overline{t})\right),$$

in which F_*_ is a normalization constant, *A* and *τ* parameterize the exponential ramp, *t* is the time since the beginning of the observations (after trimming), *x* and *y* are the positions of the trace on the detector, *m* is a slope (potentially caused by stellar variability and/or instrumental drift) and $\overline{t}$ is the average time. All parameters are given uniform priors. *τ* is required to be between 0 and 0.1, but no explicit bounds are imposed on the other parameters.

### Forward modelling

We used several forward models that take into account photochemistry to infer the properties of the atmosphere of WASP-39b from the observations. These models are based on known first-principle physics and chemistry that aid in our understanding of the important atmospheric processes at work. Also, we also use one of the models to generate a more extensive model grid to assess the atmospheric metallicity and elemental ratios of WASP-39b. These models compute the atmospheric composition by explicitly treating the thermochemical and photochemical reactions and transport in the atmosphere, and—in general—are initialized from equilibrium abundances based on a given elemental ratio, for which we scale relative to solar abundances^45^. Although the abundances of a planet’s host star are the more natural comparison point (for example, ref. ^46^), the measured multi-element abundances of WASP-39 are very nearly solar^47^. All photochemical models use the same incident stellar spectrum as that described in ref. ^4^. Finally, we also consider a radiative–convective–thermochemical equilibrium model that includes an injected SO_2_ abundance and clouds to connect oCur work to previous interpretations of near-infrared JWST spectra of WASP-39b (refs. ^2,3,15,16^).

#### VULCAN

The 1D kinetics model VULCAN treats thermochemical^48^ and photochemical^8^ reactions. VULCAN solves the Eulerian continuity equations, including chemical sources/sinks, diffusion and advection transport and condensation. We used the C–H–N–O–S network (https://github.com/exoclime/VULCAN/blob/master/thermo/SNCHO_photo_network.txt) for reduced atmospheres containing 89 neutral C-bearing, H-bearing, O-bearing, N-bearing and S-bearing species and 1,028 total thermochemical reactions (that is, 514 forward–backward pairs) and 60 photolysis reactions. The sulfur allotropes are simplified into a system of S, S_2_, S_3_, S_4_ and S_8_. The sulfur kinetics data are drawn from the NIST and KIDA databases, as well as modelling^6,49^ and ab initio calculations published in the literature (for example, ref. ^50^). The temperature-dependent ultraviolet cross-sections^8^ are not used in this work for simplicity, but preliminary tests show that their exclusion has resulted in only minor differences (less than 50% of the SO_2_ volume mixing ratio). Apart from varying elemental abundances, we applied an identical setup of VULCAN as that in ref. ^4^.

#### KINETICS

The KINETICS 1D thermo-photochemical transport model^51–54^ is used to solve the coupled Eulerian continuity equations for the production, loss and vertical diffusive transport of atmospheric species. The chemical reaction list, background atmospheric structure and assumed planetary parameters are identical to those described in ref. ^4^, except here we explore further atmospheric metallicities. Briefly, the C–H–N–O–S–Cl network used for the WASP-39b KINETICS model contains 150 neutral species that interact with each other through 2,350 total reactions, with the non-photolysis reactions being reversed through the thermodynamic principle of microscopic reversibility^55^.

#### ARGO

The 1D thermochemical and photochemical kinetics code ARGO originally used the STAND2019 network for neutral hydrogen, carbon, nitrogen and oxygen chemistry^56,57^. ARGO solves the coupled 1D continuity equation including thermochemical–photochemical reactions and vertical transport. The STAND2019 network was expanded in ref. ^58^ by updating several reactions, incorporating the sulfur network developed in ref. ^7^ and supplementing it with reactions from refs. ^59,60^, to produce the STAND2020 network. The STAND2020 network includes 2,901 reversible reactions and 537 irreversible reactions, involving 480 species composed of H, C, N, O, S, Cl and other elements.

#### EPACRIS

EPACRIS (the ExoPlanet Atmospheric Chemistry & Radiative Interaction Simulator) is a general-purpose 1D atmospheric simulator for exoplanets. EPACRIS has a root of the atmospheric chemistry model developed by Renyu Hu and Sara Seager at MIT^61–63^, and—since then—has been reprogrammed and upgraded substantially (refs. ^64,65^ and also Yang and Hu (2023), in preparation, mainly focusing on the validation of reaction-rate coefficients). We use the atmospheric chemistry module of EPACRIS to compute the steady-state chemical composition of the atmosphere of WASP-39b controlled by thermochemical equilibrium, vertical transport and photochemical processes. The chemical network applied in this study includes 60 neutral C-bearing, H-bearing, O-bearing and S-bearing species and 427 total reactions (that is, 380 reversible reaction pairs and 47 photodissociation reactions). In this chemical model, the SO_2_ volume mixing ratio is sensitive to two reactions, which are (1) H_2_S ↔ HS + H and (2) SO + OH ↔ HOSO. Briefly describing, if the HS + H → H_2_S recombination-rate coefficient is faster than 10^−11^ cm^3^ molecule^−1^ s^−1^ (the collision limit is around 10^−9^ cm^3^ molecule^−1^ s^−1^), this will result in inefficient H_2_S dissociation (that is, H_2_S starts to dissociate at higher altitude), which leads to the decreased SO_2_ formation. Unfortunately, to the best of our knowledge, there is no theoretically calculated nor experimentally measured H_2_S decomposition-rate coefficient. For this reason, in EPACRIS, we assumed that H_2_S ↔ HS + S is similar to H_2_O ↔ HO + H. However, all of the HS + H → H_2_S recombination-rate coefficients used in different models were slower than 10^−11^ cm^3^ molecule^−1^ s^−1^ and, below this range, the SO_2_ volume mixing ratio is no longer sensitive to this reaction. With regard to the SO + OH ↔ HOSO reaction, the forward reaction (barrierless reaction) is favoured at lower temperatures and higher pressures according to the HOSO potential-energy surfaces^66^. For this reason, the exclusion of this reaction from the EPACRIS chemical model shows up to two orders of magnitude increase (that is, from [SO_2_] ≈ 10^−6^ to 10^−4^) in the SO_2_ volume mixing ratio in the morning limb. However, in the evening limb, whose temperature is up to about 200 K higher compared with the morning limb, HOSO can now further dissociate to form SO_2_ and H as a result of elevated temperature, which results in the increased [SO_2_] ≈ 10^−5^ compared with the morning limb [SO_2_] ≈ 10^−6^.

#### IDIC grid

Reference ^14^ presented a grid of VULCAN photochemistry models (we term this the IDIC grid) for WASP-39b that cover a 3D volume of possible C, O and S elemental abundances without aerosols. We used these models to compare with our three spectral reductions. We fit each MIRI/LRS transmission spectrum by binning all model spectra to the regular, 0.25-μm resolution of the observed spectra, allowing for an arbitrary vertical offset for each model spectrum, and calculating *χ*^2^ for each model spectrum. We first determined the goodness of fit while holding all abundances linked to the same value (that is, C, O and S all enhanced by the same level relative to solar abundances). We fit a parabola to the three lowest *χ*^2^ points to estimate the optimal elemental abundance enhancement and its uncertainty^67^ (that is, Δ*χ*^2^ = 1). We then also compared these linked-abundance *χ*^2^ values with those derived across the entire 3D grid by allowing all three elemental abundances to vary individually. Extended Data Tables 2 and 3 show the abundances and *χ*^2^ values for these analyses.

Interpreting the spectra is challenging because the goodness of fit varies widely across the observed spectra: across all IDIC models, we find a best-fit *χ*^2^ of 14.7 for the Tiberius reduction but a best-fit *χ*^2^ of 45.4 for the Eureka! reduction (which reports much smaller measurement uncertainties). Nonetheless the linked analyses all suggest a bulk metallicity of 7.1–8.0 times solar. The standard deviation of the optimal metallicity values is 0.4, smaller than the average uncertainties in Extended Data Table 2, suggesting that the uncertainty in the bulk metallicity is dominated by statistical (or model-dependent systematic) uncertainties, rather than by differences between the several reduced spectra.

When allowing C, O and S abundances to each vary freely, in all cases, the best-fitting models show a preference for super-solar O/S ratios, sub-solar C/O and approximately solar C/S ratios. Reference ^14^ suggests that these ratios could be used to constrain the formation history of a planet by comparing with formation models^46,68^. However, a Bayesian information criterion analysis shows that, for the Tiberius and SPARTA reductions, the observed spectra do not justify the extra free parameters of numerous independent elemental abundances. The formal Bayesian information criterion value for the Eureka! reduction seems to indicate that independent abundances are justified, but this conclusion seems questionable because this spectrum gives the worst *χ*^2^ values (36.7 with just 28 data points).

#### PICASO grid

Previous observations of WASP-39b with JWST’s NIRspec PRISM, NIRISS SOSS, NIRCam F322W and NIRSpec G395H (refs. ^1–3,15,16^) were interpreted using a grid of 1D radiative–convective thermal equilibrium (RCTE) models^69^ generated with PICASO 3.0 (refs. ^70,71^). Here, to interpret the spectrum of WASP 39b observed with MIRI/LRS, we use the base clear equilibrium PICASO 3.0 version of this grid, along with a subset of the grid of PICASO 3.0 models post-processed with Virga^72,73^ to account for clouds formed from Na_2_S, MnS and MgSiO_3_. The full parameters of the original set of grids can be found in ref. ^69^. We reduced several grid points of the post-processed cloudy Virga grid. In the cloudy grid we use here, we included only one heat-redistribution factor (0.5), only one intrinsic temperature (100 K), only *f*_sed_ values ≤3 and only log_10_*K*_*zz*_ > 5, as this low of a log_10_*K*_*zz*_ is unphysically small at temperatures greater than 500 K (ref. ^74^) (for example, Fig. 2), as in the atmosphere of WASP-39b. The original grids in ref. ^69^ were only computed for wavelengths from 0.3 to 6 μm; here we extend the simulated transmission spectra of the grid out to wavelengths of 15 μm.

To assess the presence of SO_2_ in the MIRI/LRS data, we first inject a constant abundance of SO_2_ into each model at grid points of 3, 5, 7.5, 10, 20 and 100 ppm, and we then recompute the model spectra. These values of SO_2_ are therefore not chemically consistent with the rest of the atmosphere. As in the IDIC grid, we fit each transmission spectrum reduction by binning the model spectra (resampled to opacities at *R* = 20,000 (ref. ^75^)) to the resolution of the observations, allow for a vertical offset and calculate *χ*^2^ for each model spectrum. We take the top 20 best-fitting models to account for scatter in the preferred grid values and discard clear outliers.

**Extended Data Fig. 3:**
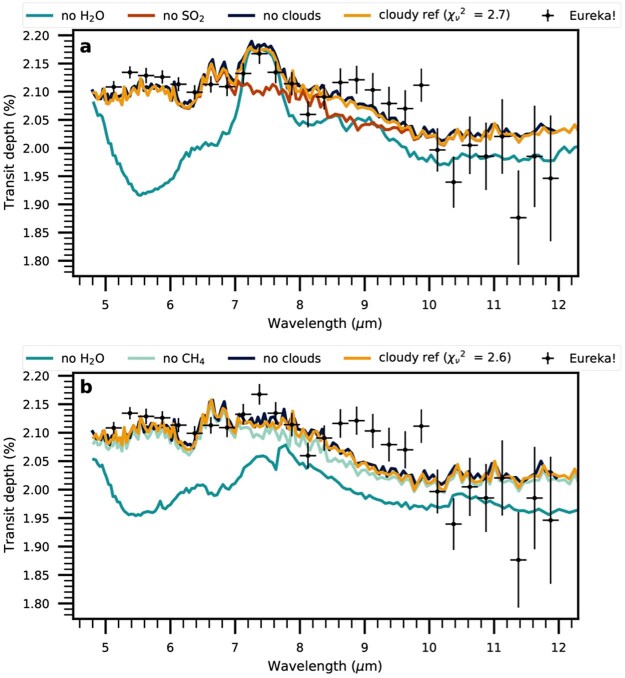
The best-fitting cloudy PICASO grid models (gold lines) are shown with and without SO_2_ compared with the JWST MIRI/LRS data (black points) from the Eureka! reduction. **a**, With SO_2_. **b**, Without SO_2_. Also shown are the best fits with H_2_O (dark teal), SO_2_ (red), CH_4_ (light teal) and clouds (navy blue) removed from the model, demonstrating which absorbers dominate the opacity of the best-fit model. When SO_2_ is not included in the model, excess CH_4_ compensates for its absorption in the Eureka! reduction, as shown in the lower panel.

Without SO_2_, although we find comparable overall fits (*χ*^2^ ≤ 2.6) to the data for the Eureka! reduction, none of the SO_2_-free RCTE models capture the rise around 7.7 or 8.5 μm. Once SO_2_ is added, we find that the overall model fit to the Eureka! reduction is slightly worse (*χ*^2^ ≤ 2.7), but the shape of the spectrum better matches at 7.7 and 8.5 μm. This slightly worse fit is driven by the slightly higher transit depths from 5 to 6 μm in the Eureka! reduction, which results in a higher baseline ‘continuum’ when SO_2_ is not included. For both the SPARTA and Tiberius reductions, the grid-model fits improve with added SO_2_. Most crucially, in the absence of SO_2_, the best-fitting clear PICASO 3.0 and cloudy PICASO 3.0 + Virga grid models across all reductions are dominated by H_2_O absorption, as well as prominent contributions from CH_4_ for the Tiberius and Eureka! data, as shown in Extended Data Fig. 3. For the Tiberius and Eureka! reductions, cloudy cases without SO_2_ result in high inferred amounts of CH_4_ (volume mixing ratio ≈ 1–50 ppm) at 10 mbar—at which the MIRI/LRS observations interrogate. These CH_4_ mixing ratios are in disagreement with the lack of CH_4_ in the atmosphere of WASP-39b observed at shorter wavelengths with NIRISS, NIRSpec and NIRCam (with best-fit models having CH_4_ volume mixing ratios of about 3 ppb, about 0.1 ppm and about 50 ppb, respectively)^2,3,15,16^. With the SPARTA reduction, rather than compensating for the lack of SO_2_ opacity with elevated CH_4_ abundances, the PICASO grid best fits invoke opacity from a high-altitude, optically thick silicate cloud.

Models with SO_2_ injected produce better overall fits to each MIRI reduction, with mixing ratios of C-bearing, O-bearing and S-bearing species in agreement with those inferred from shorter-wavelength data from NIRISS, NIRSpec and NIRCam. Therefore, our results indicate that MIRI data alone can independently constrain relevant atmospheric gaseous species. With these MIRI data, as well as the previous JWST observations, we demonstrate that SO_2_ in the atmosphere of WASP-39b is required to self-consistently interpret the data from the JWST over a wide wavelength range.

When SO_2_ is included in the RCTE PICASO 3.0 models, we find that all three reductions prefer C/O ratios less than or equal to solar values. These low C/O ratios result from the lack of methane needed to fit the data. Metallicity values range from about 10 times solar for the Eureka! and Tiberius reductions to about 10–30 times solar for the SPARTA reduction. Best fits are comparable between clear and cloudy cases, with high best-fitting values of *f*_sed_ resulting in cloud decks below the atmospheric regions examined by MIRI/LRS. The best-fitting models using MIRI therefore result in very different cloud parameters compared with models fit to shorter wavelengths^2,3,15,16^. These cloud-parameter discrepancies highlight that constraining cloud conditions requires wide wavelength coverage and may result from cloud formation localized to different atmospheric layers^20^.

Finally, within the framework of injected uniform SO_2_ abundances that do not vary with altitude, we find that all of our SO_2_ abundance grid points result in comparable model fits, preventing a strong SO_2_ abundance constraint from the PICASO 3.0 grid.

### Retrieval modelling

As well as forward modelling, we further investigated the atmosphere of WASP-39b as seen by MIRI/LRS using six different free-retrieval frameworks (see descriptions below). Free retrievals use parameterized atmospheric models to directly extract constraints on atmospheric properties from the data. Each chemical species in the model is treated as an independent free parameter, rather than abundances being calculated under assumptions such as chemical equilibrium or photochemistry. The retrievals presented in this paper all assume that the atmosphere is well mixed, so chemical abundances are held constant throughout the atmosphere. All retrievals also assume an isothermal temperature profile, as the MIRI/LRS spectrum examines a relatively small range of atmospheric pressures and, therefore, is relatively insensitive to the temperature structure. All retrievals contain some prescription for aerosols, but the details vary across the six frameworks and are described in more detail below. This variation in aerosol treatment is intentional and, by this approach, we hope to capture the impact of different retrieval choices on molecular detection and abundance measurements for MIRI. All frameworks also retrieve either a reference pressure or reference radius, to account for the so-called ‘normalization degeneracy’ (see ref. ^76^). Helios-r2 also includes the stellar radius and log(*g*), in which *g* is gravitational acceleration, as free parameters. For all frameworks, we ran the preferred model setup, and those removing H_2_O or SO_2_, allowing us to calculate their Bayesian evidence following ref. ^77^ (Extended Data Table 4).

Atmospheric models do not provide as good a match to the data at ≳10 μm, with worse fits by *χ*^2^ and *P*-value metrics than when only considering data bluewards of 10 μm. Therefore, we considered the possibility of retrieving only on the short wavelengths. Although we find that the retrieved abundances are highly sensitive to the wavelengths considered, there is no evident, data-driven argument to disregard data at longer wavelengths, and the fits are acceptable. Therefore, the atmospheric inferences presented below consider the entire MIRI/LRS spectrum from 5 to 12 μm. Further investigation into the apparent decrease in transit depth at 10 μm is warranted in future work.

#### ARCiS

ARCiS (ARtful modelling Code for exoplanet Science) is an atmospheric modelling and Bayesian retrieval package^78,79^, which uses the MultiNest^80^ Monte Carlo nested sampling algorithm to sample a parameter space for the region of maximum likelihood. ARCiS is capable of both free-molecular and constrained-chemistry (that is, assuming thermochemical equilibrium) retrievals, with the latter using GGchem^81^ for the chemistry. For this work, we use a free-molecular retrieval with a simple grey, patchy cloud model. This simple model parameterizes cloud-top pressure and the degree of cloud coverage (from 0 for completely clear to 1 for completely covered). We explored the use of a variety of molecular species in our retrievals, with most of their abundances being unconstrained by the retrieval of this dataset. In particular, we searched for further photochemical products including SO and SO_3_. The photochemical model in ref. ^4^ predicts observable amounts of SO but very little SO_3_. We find some weak-to-moderate (2.5*σ*) evidence of SO (ref. ^82^) and no evidence of SO_3_ (ref. ^83^), qualitatively matching the photochemical model predictions. Also, we find approximately 3.3*σ* evidence for the presence of a molecule such as SiH (ref. ^84^), BeH (ref. ^85^) or NO (ref. ^86^). The broad opacity features from these species, however, are indistinguishable from a continuum effect, such as haze.

In the absence of other spectral features from these molecules, and because we do not expect SiH, BeH or NO to be abundant enough (about 1,000 ppm is required, compared with a maximum of approximately 10 ppm for SiH and fractions of a ppm for BeH under the assumption of solar-abundance thermochemical equilibrium^45,81^), we exclude them in our models. We therefore present a simplified set of molecules, with only H_2_O (ref. ^22^) and SO_2_ (ref. ^23^) included, along with the parameters for the clouds. Combined with isothermal temperature and planetary radius, this totals six free parameters. The reference pressure for the radius is 10 bar. The opacities are *k*-tables from the ExoMolOP database^87^, with the line lists from the ExoMol^88^ or HITEMP^89^ database as specified. Collision-induced absorption for H_2_ and He are taken from refs. ^90,91^. We use 1,000 live points and a sampling efficiency of 0.3 in MultiNest. We used a value of 0.281*M*_J_ for the planetary mass and 0.9324*R*_⊙_ for the stellar radius.

#### Aurora

Aurora is an atmospheric inference framework with applications to transmission spectroscopy of transiting exoplanets (for example, refs. ^92,93^). The comprehensive description of the framework and modelling are explained in ref. ^94^. For this dataset, we considered a series of atmospheric models ranging from simple, cloud-free isothermal models to those with several chemical species, inhomogeneous cloud and hazes and non-isothermal pressure–temperature profiles. The parameter estimation was performed using the nested sampling algorithm^95^ through MultiNest^80^ using the PyMultiNest implementation^96^.

We find that the retrieved abundances of H_2_O and SO_2_ vary by several orders of magnitude depending on the data reduction considered, the wavelength range included (for example, above or below 10 μm) and assumptions about the atmospheric model used (for example, cloud-free versus cloudy, fully cloudy versus inhomogeneous clouds, several absorbers versus limited absorbers; see, for example, ref. ^97^).

Our initial exploration of atmospheric models finds that, when considering several species (for example, Na, K, CH_4_, NH_3_, HCN, CO, CO_2_ and C_2_H_2_), their abundances are largely unconstrained despite affecting the retrieved SO_2_ abundances by at least an order of magnitude, generally skewing them towards lower values (for example, log_10_(SO_2_) ≲ −6). The use of parametric pressure–temperature profiles (for example, ref. ^98^) do not result in substantial changes to the retrieved abundances and the resulting temperature profiles are largely consistent with isothermal atmospheres. Finally, we find that assuming cloud-free or homogeneous cloud cover can result in artificially tight constraints on the H_2_O abundances as expected (for example, refs. ^94,97,99^), motivating our choice to consider the presence of inhomogeneous clouds/hazes.

Given the above considerations, we settled on a simplified fiducial model to calculate the model preference (that is, ‘detection’; see, for example, refs. ^94,100^) for H_2_O and SO_2_, with the caveat that the retrieved abundances are highly dependent on the model/data assumptions. This simplified model only considers absorption owing to H_2_O and SO_2_ using line lists from refs. ^89,23^, respectively, H_2_–H_2_ and H_2_–He collision-induced absorption with line lists from ref. ^101^, the presence of inhomogeneous clouds and hazes following the single-sector model in ref. ^94^ (see also refs. ^99,102^) and an isothermal pressure–temperature profile. In total, our atmospheric model has eight free parameters: two for the constant-with-height volume mixing ratios of the chemical species considered, one for the isothermal temperature of the atmosphere, four for the inhomogeneous clouds and hazes and one for the reference pressure for the assumed planet radius (*R*_p_ = 1.279*R*_J_, log_10_(*g*) = 2.63 cgs, *R*_star_ = 0.932*R*_⊙_). The forward models for the parameter estimation were calculated at a constant resolution *R* = 10,000 using 1,000 live points for MultiNest.

#### CHIMERA

CHIMERA^103^ is an open-source radiative transfer and retrieval framework that has been extensively used to study the atmospheres of planetary-mass objects, ranging from brown dwarfs^104^ to terrestrial planets^105^. The forward model is coupled to a nested sampler, namely, MultiNest^80^ using the PyMultiNest^96^ wrapper. CHIMERA takes advantage of the correlated-*k* approximation^106,107^ to rapidly compute the transmission through the atmosphere. Given the flexible nature of the code, it is capable of modelling a range of different aerosol and cloud scenarios^108^, as well as a range of different thermal structures^98,109^.

For this work, we are limited to the spectral bands to which we have access, thus we only model H_2_O and SO_2_ using line data from refs. ^22,23^, respectively. We assume that the atmosphere is dominated by H_2_, with a He/H_2_ ratio of 0.1764; therefore, we also model the H_2_–H_2_ and H_2_–He collision-induced absorption^101^. We model hazes following the prescription in ref. ^110^, which treats hazes as enhanced H_2_ Rayleigh scattering with a free power-law slope. Alongside the haze calculation, we fit for a constant-in-wavelength grey cloud with opacity *κ*_cloud_. We also assess the patchiness of the cloud by linearly combining a cloud-free model with the cloudy model^111^. We find that the inclusion of hazes does not improve any of our inferences, thus our final model presented is from using the grey cloud alone. We used a value of 0.281*M*_J_ for the planetary mass and 0.932*R*_⊙_ for the stellar radius.

#### Helios-r2

Helios-r2 (ref. ^112^) (the open-source Helios-r2 code can be found at https://github.com/exoclime/Helios-r2) is an open-source, GPU-accelerated retrieval code for atmospheres of exoplanets and brown dwarfs and can be used for transmission, emission and secondary-eclipse observations (see, for example, refs. ^113–115^). It uses a Bayesian nested sampling approach to compute the posterior distributions and Bayesian evidences, based on the MultiNest library^80^.

In Helios-r2, the chemical composition can be constrained assuming chemical equilibrium using the FastChem (the open-source FastChem code can be found at https://github.com/exoclime/FastChem) chemistry code^116,117^ or by performing a free abundance retrieval with either isoprofiles or vertically varying abundances. The temperature profile can also be either described by an isoprofile or allowed to vary with height by using a flexible description based on piece-wise polynomials or a cubic spline approach. Given the limited number of available observational data points in this study, we chose to describe the temperature and the chemical abundances with isoprofiles.

In our final retrieval calculations, only two gas-phase species are directly retrieved (H_2_O and SO_2_), whereas H_2_ and He are assumed to form the background atmosphere based on their solar H/He ratio. Further chemical species, such as HCN, CO, CO_2_ or CH_4_ for example, were tested but resulted in unconstrained posteriors.

We used the ExoMol POKAZATEL line list for H_2_O (ref. ^22^) and the ExoAmes SO_2_ (ref. ^23^) line list in our retrievals. Line list data for HCN, CO and CH_4_ were taken from refs. ^118–120^, respectively. The opacities were calculated with the open-source opacity calculator HELIOS-K (refs. ^121,122^) (the open-source HELIOS-K code can be found at https://github.com/exoclime/HELIOS-K) and are available on the DACE platform (https://dace.unige.ch). The collision-induced absorption of H_2_–H_2_ and H_2_–He pairs was taken from refs. ^123–125^.

In the retrieval calculations, we added a grey-cloud layer with the top pressure of the cloud as a free parameter. Furthermore, we used the surface gravity and the stellar radius as free parameters with Gaussian priors based on their measured values to incorporate their uncertainties in the retrieval results.

For the retrieval calculations in this study, 2,000 live points and a sampling efficiency of 0.3 for an accurate determination of the Bayesian evidence were used.

#### NEMESIS

NEMESIS^126^ is an open-source retrieval algorithm that allows simulation of a range of planetary and substellar bodies, using either nested sampling^95,127^ or optimal estimation^128^ to iterate towards a solution. It has been used extensively to model the atmospheres of transiting exoplanets (for example, ref. ^99^). NEMESIS uses the correlated-*k* approximation^106^ to allow rapid calculation of the forward model. It allows flexible parameterization of aerosols and gas abundance profiles and can also be used to simultaneously and consistently model several planetary phases (for example, ref. ^129^).

In this work, we use the nested sampling algorithm PyMultiNest^80,96^, with 2,000 live points. We include H_2_O line data from the POKAZATEL line list^22^ and SO_2_ line data from the ExoAmes line list^23^, using *k*-tables calculated as in ref. ^87^. Collision-induced absorption information for H_2_ and He is taken from refs. ^90,91^. Aerosol is modelled as an opaque grey cloud deck, with a variable top pressure. We also retrieve a fractional cloud-coverage parameter, simulating the total terminator spectrum as a linear combination of a cloudy spectrum and an otherwise identical clear spectrum. We also tested the inclusion of a simple haze model with a tunable scattering index parameter, after refs. ^102,99^, but found that the retrieved scattering index gave an unrealistically steep spectral slope. We therefore present the models including only a grey cloud deck. We used a value of 0.281*M*_J_ for the planetary mass and 0.9324*R*_⊙_ for the stellar radius.

#### Pyrat Bay

Pyrat Bay^130^ (the PYthon RAdiative-Transfer in a BAYesian framework) is an open-source software that enables atmospheric forward and retrieval modelling of exoplanetary spectra^131^. This software uses parametric temperature, composition and altitude profiles as a function of pressure to generate emission and transmission spectra. The radiative-transfer model considers various sources of opacity, including alkali lines^132^, Rayleigh scattering^110,133^, ExoMol and HITEMP molecular line lists^89,134^, collision-induced absorption^90,91^ and cloud opacities. To optimize retrieval, Pyrat Bay compresses these large databases while retaining essential information from dominant line transitions, using the method described in ref. ^135^. The software offers various cloud-condensate prescriptions, including the classic ‘power law + grey’ model, a ‘single-particle-size’ haze profile, a ‘patchy-clouds’ model with partial coverage factor^136^ and a complex parameterized Mie-scattering thermal-stability model (J.B. et al., manuscript in preparation and refs. ^137,138^). Furthermore, Pyrat Bay allows users to adjust the complexity of the compositional model, ranging from a ‘free-retrieval’ approach in which molecular abundances are freely parameterized to a ‘chemically consistent’ retrieval that assumes chemical equilibrium. For the chemically consistent retrieval, users can choose between the numerical TEA code^139,140^ and the analytical RATE code^141^, both of which can rapidly calculate volume mixing ratios of desired elemental and molecular abundances across a wide range of chemical species. The software also provides a variety of temperature models, including isothermal profiles and physically motivated parameterized models (for example, refs. ^98,109^). To sample the parameter space and perform Bayesian inference, Pyrat Bay is equipped with two Bayesian samplers: the differential-evolution Markov chain Monte Carlo algorithm^142^, implemented following ref. ^143^, and the nested sampling algorithm, implemented using PyMultiNest^80,96^. These algorithms use millions of models and thousands of live points to explore the parameter space effectively.

For this analysis, we conducted a free retrieval and tested various model assumptions. These involved testing all temperature parametrizations implemented in our modelling framework, a wide range of chemical species opacities expected to exhibit observable spectral features in the MIRI wavelength region, H_2_O (ref. ^22^), CH_4_ (ref. ^144^), NH_3_ (refs. ^145,146^), HCN (refs. ^118,147^), CO (ref. ^119^), CO_2_ (ref. ^89^), C_2_H_2_ (ref. ^148^), SO_2_ (ref. ^23^), H_2_S (ref. ^149^) and different cloud prescriptions. Our transmission spectrum was generated at a resolution of *R* ≈ 15,000 and then convolved to match the MIRI resolution of 100. We assumed a hydrogen-dominated atmosphere with a He/H_2_ ratio of 0.1764 and accounted for H_2_–H_2_ (ref. ^90^) and H_2_–He (ref. ^90^) collision-induced absorptions. We used the same values of the stellar radius and planetary mass as the NEMESIS pipeline. To evaluate the likelihood of our models, we used the PyMultiNest algorithm with 2,000 live points. Similar to the findings of other retrieval frameworks, most of the considered species were largely unconstrained. The Mie-scattering cloud models did not detect spectral signatures of any condensates in the data, and the more complex temperature models yielded temperature profiles that were largely consistent with an isothermal atmosphere. Only H_2_O and SO_2_ exhibited detectable spectral features in the data and the assumption of a patchy grey cloud was the most suitable for the quality of the observations. Our final atmospheric model, applied to the reduction data of each team, consisted of six free parameters: two for the constant-with-height volume mixing ratios of the chemical species, one for the isothermal temperature of the atmosphere, one for the planetary radius and two for the patchy opaque cloud deck.

#### TauREx

TauREx (Tau Retrieval for Exoplanets) is an open-source, fully Bayesian inverse atmospheric retrieval framework^150,151^. We adopted the latest version (3.1) of the TauREx software^152,153^. This version makes exclusive use of absorption cross-sections, as the correlated-*k* tables are no longer computationally advantageous^152^. We selected the PyMultiNest algorithm to sample the parameter space^80,96^. The atmosphere was modelled with 200 equally spaced layers in log pressure between 10^6^ and 10^−4^ Pa. In all our tests, we assumed an isothermal profile and constant mixing ratios with altitude. The radiative-transfer model accounts for absorption from chemical species, collision-induced absorption by H_2_–H_2_ and H_2_–He (refs. ^123–125^) and clouds. We performed initial retrieval tests including a long list of molecular species, H_2_O (ref. ^22^), SO_2_ (ref. ^23^), CO (ref. ^119^), CO_2_ (ref. ^89^), CH_4_ (ref. ^120^), HCN (ref. ^154^), NH_3_ (ref. ^155^), FeH (ref. ^156^) and H_2_S (ref. ^149^), but found that only H_2_O and SO_2_ may have detectable features in the observed MIRI spectra. We validated statistically the detection of both H_2_O and SO_2_ by comparing the Bayesian evidence of best-fit retrievals with both species versus those obtained by removing either molecule. We considered the following scenarios: (1) a clear atmosphere; (2) an atmosphere with an optically thick cloud deck, for which we fitted the top-layer pressure; and (3) an atmosphere with haze, using the formalism of ref. ^157^ for modelling the Mie scattering. Finally, we selected the retrievals with a thick cloud deck, which provide the most consistent scenarios across data reductions, and with slightly more conservative error bars. Only for the Eureka! reduction was the haze model slightly favoured (2.4*σ*), but the corresponding molecular abundances are affected by strong degeneracy between water and haze. For other reductions, the inferred molecular abundances are essentially independent of the retrieval scenario. We used a value of 0.281*M*_J_ for the planetary mass and 0.939*R*_⊙_ for the stellar radius.

#### Free-retrieval results

**Extended Data Fig. 4:**
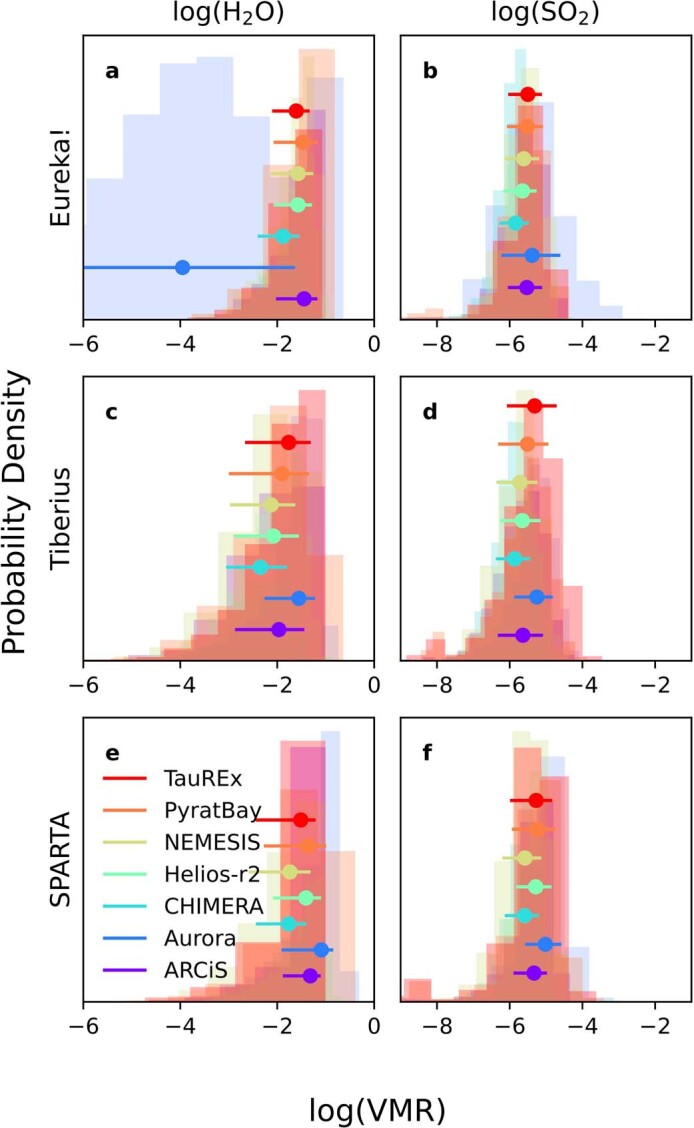
Retrieved log of SO_2_ and H_2_O volume mixing ratio posteriors from all six retrieval codes and three data reductions. Median values and 1*σ* uncertainties are given by the coloured points. VMR, volume mixing ratio.

The results from all retrieval frameworks, across all three reductions, are presented in Extended Data Table 4 and shown in Extended Data Fig. 4. These serve to illustrate the general consistency of the results for SO_2_ and H_2_O, whilst also highlighting the differences in retrieved abundance for some cases. We reiterate that the different retrieval teams made a variety of choices in the setup of their retrievals, which are described in more detail above. The overall good agreement is testament to the robustness of our detection of SO_2_ in the MIRI dataset.

We recover a range of median abundances for log(SO_2_) of between −5.9 and −5.0 across all reductions and retrieval frameworks. The overall spread of log(SO_2_) across all retrievals and reductions, from the lowest −1*σ* bound to the highest +1*σ* bound, is −6.4 to 4.6 (the range reported in the main text refers only to the retrievals on the Eureka! reduction), corresponding to volume mixing ratios of 0.4–25 ppm (0.5–25 ppm if only retrievals on the Eureka! reduction are considered). Note that this range could potentially be wider if a more extensive exploration of possible cloud and haze configurations were conducted, which we leave to future work.

SO_2_ is detected at more than 3*σ* significance in all cases except the Helios-r2 retrievals for Eureka! and SPARTA (2.54*σ* and 2.99*σ*, respectively) and the Aurora retrieval for SPARTA (2.95*σ*). The Helios-r2 model has the simplest representation of clouds but also allows the stellar radius and planetary log(*g*) to vary, so it is likely that the precise combinations of the Eureka! and SPARTA spectra and the chosen variables result in weaker detections for SO_2_, because other parameters have more freedom to compensate for a lack of SO_2_ in this framework. Similarly, the Aurora framework has a unique representation of aerosol, including both cloud and haze, with the cloud-top pressure as a free parameter. This also increases the flexibility of the model to compensate for changes in the SO_2_ abundance. In summary, free retrievals provide a broadly consistent picture, which is also consistent with the SO_2_ volume mixing ratios from the best-fitting photochemical models (see, for example, Fig. 4).

Test runs with the ARCiS retrieval also included SO opacity, which was not included in the other retrieval schemes. The existence of SO is not ruled out by these retrievals, with weak-to-moderate (2.5*σ*) evidence for it being present in the atmosphere. If present, it contributes to the spectrum at around 9 μm and is an extra source of opacity overlapping with the longer-wavelength end of the broad SO_2_ feature. The presence of SO is consistent with photochemical predictions and should be an avenue for future exploration.

We also retrieve log(H_2_O) abundances in all cases. Mostly, the median values for nearly all retrievals and reductions range from log(H_2_O) of −2.3 to −1.1, with an anomalously low value for the Eureka! reduction and the Aurora (−3.9) retrieval. This retrieval framework includes haze, so we postulate that—in this case—the haze slope is compensating for the shape of the H_2_O feature. Although the CHIMERA retrieval also includes haze and cloud, the cloud is uniformly distributed and the opacity is scaled, whereas Aurora has the cloud-top pressure as a free parameter. This probably accounts for the different solutions between these two codes. The Eureka! reduction also results in a spectrum with a slightly smoother downward slope between 5.2 and 6.5 μm than the other two reductions, which contributes to the preference for haze over H_2_O absorption in the Aurora retrieval.

The main H_2_O absorption feature in the MIRI/LRS range is a broad feature centred around 6 μm, but extending beyond the short-wavelength cut-off and also into the region affected by SO_2_. Slight differences in the shape of the spectrum between the three reductions at the shortest wavelengths, which is the region most sensitive to H_2_O, drive the subtle differences in the retrieved H_2_O abundances between those reductions. Eureka! and SPARTA have very similar transit depths and yield slightly larger H_2_O abundances (range excepting outliers: −1.9 to −1.1) than the Tiberius reduction (range: −2.3 to −1.5).

Although all retrievals include some prescription for cloud and/or haze, the parameters are generally poorly constrained. For ARCiS, CHIMERA and Pyrat Bay, no meaningful constraints on any cloud properties were obtained for any reductions. For Helios-r2, 1*σ* lower limits on log(cloud-top pressure) in bar of −1.85, −1.62 and −1.78 are found for the Eureka!, Tiberius and SPARTA reductions, respectively. Similarly, TauREx provides 1*σ* lower limits on log(cloud-top pressure) of −1.60, −1.97 and −2.03 for Eureka!, Tiberius and SPARTA, respectively. For NEMESIS, we find that the cloud-top pressure and cloud fraction are degenerate, but high cloud fractions with low cloud-top pressures are not permitted, so we can rule out high, opaque cloud covering a large percentage of the terminator. For Aurora/Eureka!, the haze-scattering slope is constrained to $\gamma =-{4.6}_{-1.8}^{+1.0}$, consistent with a Rayleigh-scattering slope (*γ* = −4) within 1*σ*. In summary, we can rule out a grey cloud extending to low pressures with broad terminator coverage, but otherwise with such varied results across reductions and retrievals, we cannot place any constraints on cloud or haze properties.

## Online content

Any methods, additional references, Nature Portfolio reporting summaries, source data, extended data, supplementary information, acknowledgements, peer review information; details of author contributions and competing interests; and statements of data and code availability are available at 10.1038/s41586-024-07040-9.

## Figures and Tables

**Extended Data Table 1:**
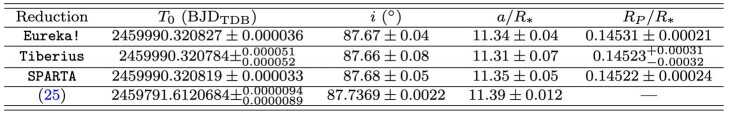
The system parameters resulting from the white-light-curve fits

The system parameters for each reduction pipeline as well as the parameters from a joint consideration of all observing wavelengths are reported.

**Extended Data Table 2:**
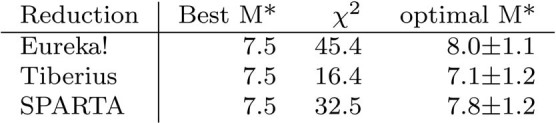
The best-fit planet metallicity relative to solar from the IDIC grid

These results from the IDIC grid assume that C, O and S have the same abundance enhancement relative to solar (that is, M*).

**Extended Data Table 3:**
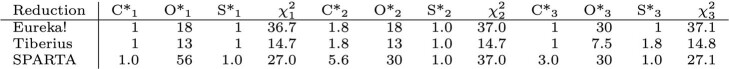
The best-fit elemental abundances from the IDIC grid

These results from the IDIC grid assume that C, O and S can take different abundances relative to solar (that is, C*, O*, S*). *χ*^2^ for the three best-fitting model spectra for each of the three reductions are shown.

**Extended Data Table 4:**
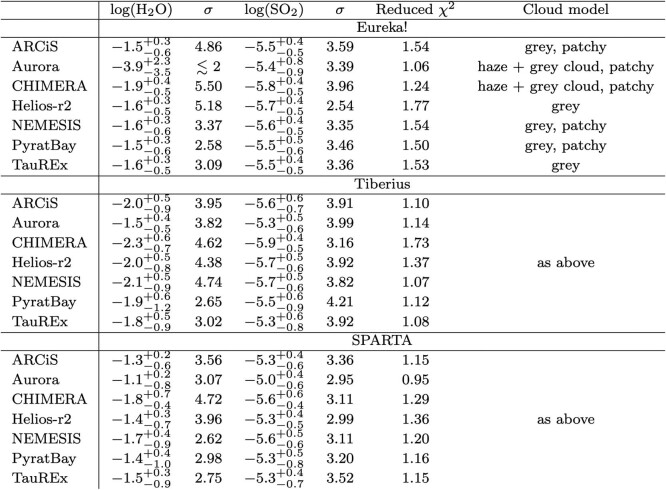
The free-retrieval results for H_2_O and SO_2_ volume mixing ratios

These results include the detection significance and the goodness of fit for each individual retrieval. This table collects all of the free-retrieval results for H_2_O and SO_2_ volume mixing ratios, together with their detection significance and the goodness of fit for each individual retrieval. The cloud model used for each retrieval code is also noted. For the most part, the abundances are consistent between retrieval codes for a given reduction, although there is some variation between reductions.

## Data Availability

The data used in this paper are associated with JWST programme DD-2783 and are available from the Mikulski Archive for Space Telescopes (https://mast.stsci.edu). The data products required to generate Figs. 1–4 and Extended Data Figs. 1–4 are available at 10.5281/zenodo.10055845. All further data are available on request.

## Code availability

The codes VULCAN and gCMCRT used in this work to simulate composition and produce synthetic spectra are publicly available: VULCAN^8,48^ (https://github.com/exoclime/VULCAN); gCMCRT^158^ (https://github.com/ELeeAstro/gCMCRT). The SPARTA software to reduce JWST MIRI and NIRCam time-series spectra is publicly available: SPARTA^44^ (https://github.com/ideasrule/sparta). The Tiberius software to reduce and analyse JWST time-series spectra is publicly available: Tiberius^38,40^ (https://github.com/JamesKirk11/Tiberius). Six of the free-retrieval codes are available at the following locations: ARCiS (https://github.com/michielmin/ARCiS); CHIMERA (https://github.com/mrline/CHIMERA); Helios-r2 (https://github.com/exoclime/Helios-r2); NEMESIS (https://github.com/nemesiscode/radtrancode); Pyrat Bay (https://github.com/pcubillos/pyratbay); TauREx (https://github.com/ucl-exoplanets/TauREx3_public). The Eureka! analyses used the following publicly available codes to process, extract, reduce and analyse the data: STScI’s JWST calibration pipeline^26^, Eureka!^24^, starry^29^, PyMC3 (ref. ^32^) and the standard Python libraries numpy^159^, astropy^160,161^ and matplotlib^162^.
